# Assessment of PD-1 and PD-L1 tissue expression levels in lichen planus patients: a case–control study

**DOI:** 10.1007/s00403-024-02838-z

**Published:** 2024-03-02

**Authors:** Maha Fathy Elmasry, Rana Ahmed Mosaad, Omar Ahmed Azzam, Laila Ahmed Rashed, Aya Fahim

**Affiliations:** 1 Dermatology Department, Faculty of Medicine, Cairo University, Cairo, Egypt; 2 Medical Biochemistry and Molecular Biology Department, Faculty of Medicine, Cairo University, Cairo, Egypt

**Keywords:** Lichen planus, Programmed cell death protein-1 (PD-1), Programmed cell death ligand-1 (PD-L1)

## Abstract

Programmed cell death protein-1 (PD-1) is an immune checkpoint protein, PD-1 interaction with PD ligand-1 (PD-L1) is essential for maintaining immunological tolerance. The study aimed to study and compare the levels of PD-1 and PD-L1 in lesional and nonlesional skin of lichen planus (LP) patients and compare these levels to normal healthy controls to assess their role in the pathogenesis of LP. This case–control study involved 30 patients with LP and 30 healthy age-and sex-matched controls. After clinical assessment of the severity by LP severity index score (LPSI), skin biopsies were taken from lesional and nonlesional skin of LP patients and from normal skin in healthy controls for assessment of the tissue levels of PD-1 and PD-L1 by ELISA. The tissue levels of both PD-1 and PD-L1 were significantly higher in healthy controls than in both lesional and nonlesional skin of LP patients (*P* < 0.001). Also, significantly higher PD-l and PD-L1 levels in nonlesional skin than in lesional skin of LP patients were reported (*P* < 0.001). No significant correlations were found between lesional and nonlesional PD-1, PD-L1 levels, or LPSI score. Based on the fact that PD-1/PD-L1 interaction is important to maintain tolerance and protection against autoimmune diseases, in addition to our study results that revealed lower levels of PD-1/PD-L1 in LP skin than in healthy skin, we can conclude that PD-1/PDL-1 may be incriminated in the pathogenesis of LP. ClinicalTrials.govID: NCT04892381.

**Supplementary Information:**

The online version contains supplementary material available at 10.1007/s00403-024-02838-z.

## Introduction

Lichen planus (LP) is a relatively common chronic inflammatory disease of unknown etiology [1]. The prevalence of cutaneous LP worldwide range between 0.2 and 1.0% of the adult population, and it is outnumbered by oral LP (OLP) in most study populations [2].

Although the pathogenesis of LP is still obscure, the present immunological understanding categorizes it as an interferon-gamma (IFN-γ)-driven disorder, thus defined as a T-helper cell-1 (Th-1) dominated disorder [3].

The immunological checkpoints are regulators of immunological tolerance by making the immune cells unresponsive to self-antigens, and protect against autoimmunity through multiple co-stimulatory and co-inhibitory receptors [4].

The programmed cell death protein-1 (PD-1) receptor is one of the co-inhibitory receptors, expressed on T-lymphocytes including the helper type, cytotoxic, regulatory T-lymphocytes (Tregs), macrophages, and other inflammatory cells [5]. Its expression is upregulated on T-cells after being activated by the T-cell receptors [6].

There are two ligands for the PD-1 receptor; PD-L1 which is expressed on T-cells, B-cells, dendritic cells (DCs), macrophages, and other non-hematopoietic cells, and PD-L2 whose expression is restricted to the activated DCs, macrophages, bone marrow-derived mast cells, and peritoneal B-cells. The interaction between PD-1 with its ligands limits T-cells activation [7].

Although the interaction between PD-L2/PD-1 exhibits higher affinity compared to PD-1/PD-L1, PD-L2 expression is lower than PD-L1, so PD-L1 is favored as a primary ligand for PD-1 [8].

There is accumulating evidence that different types of tumors including melanoma exploit PD-1-dependent immunosuppression for immune evasion, by inducing apoptosis of tumor-specific T-cells. High expression of PD-L1 on tumor cells correlates with unfavorable prognosis [9].

Based on the hypothesis that interruption of PD-1/PD-L1 binding may activate the tumor-specific T-cells, several types of immune checkpoint inhibitors (ICI) targeting PD-1 or its ligand have been approved for various cancer therapies [10].

Cutaneous immune-mediated adverse events that mimic, trigger, or exacerbate autoimmune skin diseases namely LP, vitiligo, psoriasis, and bullous pemphigoid (BP) occur in 30–40% of patients receiving ICI [10–14]. Previously published studies have assessed the expression of PD-1 protein levels in different cutaneous diseases including psoriasis, vitiligo, pemphigus vulgaris, oral LP, and mycosis fungoides [15–18].

In an attempt to clarify the role of the PD-1/PD-L1 pathway in the pathogenesis of cutaneous LP and based on the hypothesis that a reduction of PD-1 and PD-L1 expression could be associated with a high cytotoxic immune response and development of cutaneous LP especially in clinically more severe LP cases, this study was formulated.

## Subjects and methods

This case–control study included 30 patients with LP and 30 sex-and age-matched healthy individuals (non-relatives of the patients) who served as the control group, all were recruited from the dermatology outpatient clinic at Cairo University Hospital.

The protocol of this case–control study was revised and approved by the Research Ethics Committee of the Faculty of Medicine. ClinicalTrials.govID: NCT04892381.

The study was conducted during the period between May 2021 and October 2022. Patients enrolled in this study were above 18 years old, had the classic type of cutaneous LP, and had not received any topical medications for 2 weeks or systemic medications for at least 4 weeks before they participated in the study.

Excluded patients were those with other types of LP than the classic form, mucosal LP, and lichenoid drug eruption, in addition to any patient with any other skin or systemic diseases that may affect the PD-1 levels such as cancers including skin and non-skin cancers.

An informed written consent for participation, photography, and publication, was signed by all included patients.

### Patients’ assessment

All patients were subjected to a detailed history including age, occupation, onset, course, duration of the disease, past medical treatment history, and any associated skin or systemic diseases.

Total body skin examination was done including an assessment of the disease severity by using LP Severity Index (LPSI) [19]. The details of LPSI are illustrated in the supplementary file (Appendix S1).

### Methodology

Punch skin biopsies (3mm Advin Health Care Manufacturer) were taken from both the LP lesion and the unaffected skin (5mm apart from the edge of the lesion) from sun-covered sites, and control skin biopsies (3mm) were taken during their plastic surgery. Skin biopsy specimens were stored frozen at -80°C until examined.

Tissue levels of PD-1 and PD-L1 were measured by enzyme-linked immunosorbent assay (ELISA) using human PD-1 ELISA Kit (Catalog Number: ELK4353), for the quantitative estimation of endogenic human PD-1 concentrations in tissue homogenates and human PD-L1 ELISA Kit (Catalog Number: ELK3055) for PD-L1 concentrations in tissue homogenates. The details of the laboratory steps are illustrated in the supplementary file (Appendix S1).

### Statistical methods

The details of the statistical methods are illustrated in the supplementary file (Appendix S1).

## Results

The demographic data of both cases and controls showed no significant difference concerning age and gender. Both the demographic data and clinical features of the patients (sex, age, past medical history, duration of the disease, and LPSI score) and controls (sex and age) are listed in Table 1.

**Table 1:** Demographic and clinical data of patients and controls

	LP patients	Controls	*P* value*
Age (Mean ± SD)	36.30 ± 12.74	36.57 ± 12.38	0.970
Gender (Count (%))			
M	11(36.7%)	12(40.0%)	0.791
F	19(63.3%)	18(60.0%)	
LPSI (Mean ± SD)	20.37 ± 11.35		
Duration in months (Mean ± SD)	2.71 ± 2.12		
Short duration (≤ 2 months) (Count (%))	17(56.7%)		
Long duration (˃2 months) (Count (%))	13(43.3%)		

*LP* lichen planus, *M* males, *F* females, *SD* standard deviation, *LPSI* lichen planus severity index score, *P value is significant if < 0.05

### PD-1 and PD-L1 levels

**Fig. 1:**
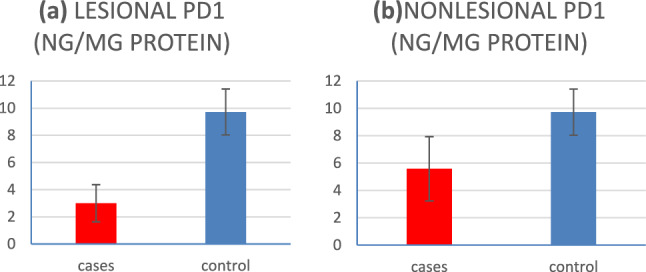
**a** PD-1 skin levels in lesional skin of LP patients versus controls, **b** PD-1 skin levels in nonlesional skin of patients versus controls

**Fig. 2:**
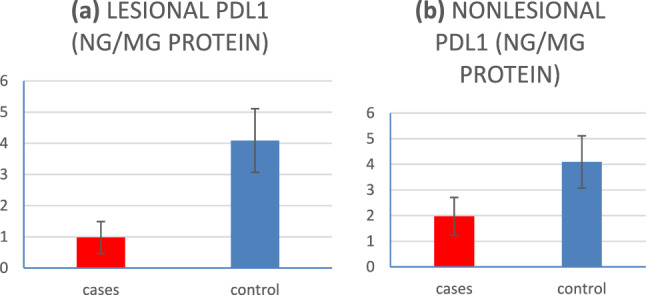
**a** PD-L1 skin levels in lesional skin of LP patients versus controls, **b** PD-L1 skin levels in nonlesional skin of patients versus controls

**Fig. 3:**
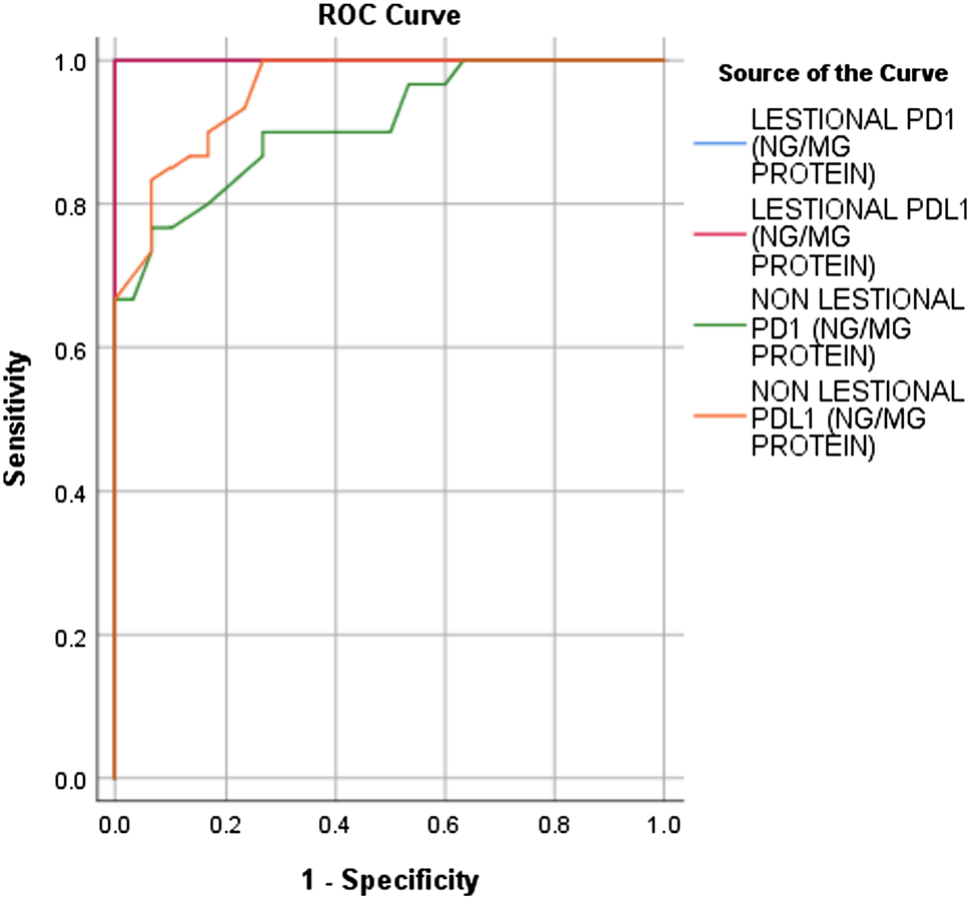
Ninety-five% confidence intervals of PD-1/PD-L1 in LP patients in lesional and nonlesional skin of patients and controls

Significantly higher PD-1 and PD-L1 levels were detected in controls compared to lesional and nonlesional skin of LP patients (*P* < 0.001), as shown in Table 2 (Figs.1, 2). Ninety-five% confidence intervals and cutoff values of PD-1/PD-L1 in LP patients’(lesional and nonlesional) skin are listed in Table 2 (Fig. 3).

**Table 2:** PD-1 and PD-L1 levels in LP patients and controls

	LP patients	95% Confidence interval			Control	*P* value
	Mean ± SD	Lower bound	Upper bound	Cutoff level	Mean ± SD	
Lesional PD-1 (ng/mg protein)	3.00** ± **1.37	1.000	1.000	< 6	9.72** ± **1.69	< 0.001*
Nonlesional PD-1 (ng/mg protein)	5.58** ± **2.35	0.840	0.983	< 7.35	9.72** ± **1.69	< 0.001*
*P* value	< 0.001*					
Lesional PD-L1 (ng/mg protein)	0.98** ± **0.51	1.000	1.000	< 2.15	4.09** ± **1.02	< 0.001*
Nonlesional PD-L1 (ng/mg protein)	1.97** ± **0.74	0.917	1.000	< 2.75	4.09** ± **1.02	< 0.001*
*P* value	< 0.001*					

*LP*: lichen planus, *SD*: standard deviation, *PD-1*: programmed cell death protein-1, *PD-L1*: Programmed cell death ligand-1,*P < 0.001 is highly significant

Significantly higher PD-1 and PD-L1 levels were detected in nonlesional skin compared to lesional skin of LP patients (*P* < 0.001) as shown in Table 2. Significantly higher PD-1 and PD-L1 levels were also found in nonlesional skin in both short duration (≤ 2 months) and chronic (˃2 months) disease compared to lesional PD-1 and PD-L1 levels (*P* < 0.001) as shown in Table 3.

**Table 3:** Comparison between short duration and chronic disease as regard the levels of both PD-1 and PD-L1 in either lesional or nonlesional skin of LP patients

		Short duration (n = 17)	Chronic disease (n = 13)	*P* value*
PD-1 (Mean ± SD)	Lesional skin (ng/mg protein)	3.26 ± 1.47	2.66 ± 1.21	0.213
	Nonlesional skin (ng/mg protein)	5.81 ± 2.29	5.27 ± 2.48	0.621
	*P* value	< 0.001**	< 0.001**	
PD-L1 (Mean ± SD)	Lesional skin (ng/mg protein)	1.11 ± 0.59	0.82 ± 0.32	0.245
	Nonlesional skin (ng/mg protein)	2.08 ± 0.83	1.84 ± 0.59	0.509
		< 0.001**	< 0.001**	

*SD* standard deviation, *PD-1* programmed cell death protein-1, *PD-L1* Programmed cell death ligand-1, *P ˃ 0.05 is non-significant, **P < 0.001 is highly significant

However, no statistically significant differences were found upon comparing the short duration and chronic disease as regard the levels of both PD-1 and PD-L1 in either lesional or nonlesional skin of LP patients (*P* > 0.05) as shown in Table 3.

### PD-1 and PD-L1 correlations to demographic and clinical data in LP patients and controls

Significantly negative correlations (*P* < 0.001) were detected between both PD-1/PD-L1 in the skin of LP patients (in both lesional and nonlesional skin) and age of the patients (r = − 0.863, − 0.656, − 0.713, and − 0.603 respectively), while no correlations between either PD-1 or PD-L1 and age of controls were found (*P* > 0.05).

No correlations were found between PD-1/PD-L1 and the gender of either the patients or the control groups (***P*** > 0.05). No correlations were found between the LPSI and either PD-1 or PD-L1 levels (in lesional or nonlesional skin). Also, no correlation was found between LPSI and the duration of the disease (*P* > 0.05).

### PD-1 and PD-L1 correlations

Significantly positive correlations were found between PD-1 and PD-L1 (*P* < 0.001) in both lesional and nonlesional skin (r = 0.885 and 0.713 respectively). Also, a significantly positive correlation was found between lesional PD-1 and nonlesional PD-1 levels (*P* < 0.001, r = 0.758) as well as between lesional PD-L1 and nonlesional PD-L1 levels (*P* < 0.001, r = 0.892). No correlation was found between the level of PD-1 and PD-L1 in controls (*P* > 0.05).

## Discussion

Although the exact pathogenesis of LP has not been fully elucidated, it is considered a chronic inflammatory autoimmune skin condition caused by a complex interplay between genetic, environmental, and immunological factors. Recent data indicate the role of antibodies in LP pathogenesis [20].

Environmental factors such as viral infections (hepatitis C virus especially), drugs, vaccines, and metals could trigger the appearance of LP in patients with genetic background of LP (evidenced by the familial cases of LP and the occurrence in monozygotic twins) [2].

A cell-mediated immune response is fundamental in LP pathogenesis, with dominant cytotoxic (CD8 + ve) T-cell infiltration complemented by the action of the Th1 and interleukin (IL)-23/Th-17 axis with predominant release of IFN-γ, therefore LP is considered as a Th-1 dominated disorder [21].

The immune checkpoints, PD-1 and its ligands, are widely expressed and exert an immunoregulatory role in T-cell activation and tolerance [22]. Cross-linking of PD-1 by its ligands; PD-L1 and PD-L2, leads to the suppression of T-cell responses by promoting apoptosis [23].

In the treatment of different cancers, PD-1 blockade was found to shift the immune response toward a proinflammatory Th1/Th17 response, as evidenced by increased levels of IFN-γ, tumor necrosis factor-alpha (TNF-α), IL-2, IL-6, and IL-17A which are the main players in the pathogenesis of different autoimmune skin diseases including LP, and to reduce the production of IL-5 and IL-13 by the Th2 cells [24].

Supported by the appearance of immunological cutaneous adverse events such as LP and lichenoid eruption after using PD-1 inhibitors in the treatment of cancers [14], all these findings call for a deeper investigation of the role of PD-1/PD-L1 levels in LP pathogenesis.

In the present study, the healthy controls had significantly higher levels of both PD-1 and PD-L1 compared to LP patients (in both lesional and nonlesional skin). Also, a significantly higher PD-1 and PD-L1 levels were detected in nonlesional skin compared to lesional skin of LP patients.

Moreover, there was a significant positive correlation between lesional and nonlesional skin levels of PD-1 and also PD-L1, in addition to the positive correlation between PD-1 and PD-L1 in lesional and nonlesional skin of LP patients.

Based on the fact that PD-1/PD-L1 interaction inhibits lymphocyte activation, the study results support the hypothesis that decreased expression of PD-1 and PD-L1 in LP patients could accelerate lymphocyte infiltration and cause failure to regulate CD8 + T-lymphocytes targeting the epidermis in the pathogenesis of LP and also low PD-L1 expression may accelerate macrophage infiltration [25].

This is the first study as far as we know to evaluate the role of PD-1 and PD-L1 pathway in cutaneous LP patients' lesional and nonlesional skin and to compare it with healthy controls, also the correlation between lesional and nonlesional PD-1 and PD-L1 was not done before in patients with LP.

In support of the present study, it has been shown that PD-1 and PD-L1 expression levels were minimal in 12 patients with LP compared to 12 patients with erythema multiforme in a study done by Shirouchi et al. [25]. Moreover, Costa et al. [4] reported reduced/absent expression of PD-L1 with lower levels of PD-1 + T-lymphocyte cells in the OLP patients. The results of the present study suggest that the changes in the levels of the PD-1/PD-L1 pathway may have a role in LP pathogenesis.

On the contrary, a study done by Zhou et al. [26] found that PD-1 and PD-L1 expressions were increased in peripheral blood T-cells from OLP patients with a positive correlation between PD-L1 expression and the severity of OLP. However, the study was done on OLP cases only and they assessed the levels of PD-1 and PD-L1 on peripheral blood T-cells without assessment of tissue levels of lesional and nonlesional PD-1 and PD-L1.

On the other hand, studies showed that although LP, psoriasis, and vitiligo differ in how they induce the immune system, they share many similarities in their pathogenesis with dominant Th1/IFN-γ and TNF-α cytokine profiles [27].

The key player leading to inflammatory responses and tissue damage in these diseases is IFN- γ by increasing the sensitivity of keratinocytes (KCs) and cytotoxic T lymphocytes activation through enhancing major histocompatibility (MHC) class I expression, and upregulating DCs MHC class II expression which helps the antigen presentation to CD4 + T-cells, while TNF-α cytokine acts in synergism with IFN-γ on the destruction of KCs and melanocytes [28–30].

The main contributors in the pathogenesis of LP, psoriasis, and vitiligo are T lymphocytes either helper or cytotoxic. The activation of the helper group of T lymphocytes leads to epithelial damage and destroys the extracellular matrix by the released cytokines and matrix metalloproteinases, while the cytotoxic group through FAS-FASL and perforin/granzyme pathways act by inducing apoptosis in KCs, melanocytes, and epithelial basal cell layer [20].

Supporting the present study, a previous study by Kim et al. [31] revealed that significantly low levels of PD-L1 and PD-L2 in psoriasis when compared to the healthy controls, however, the level of PD-1 was not assessed in this study.

In addition, another study done by Nagui et al. [16] showed that the PD-1 tissue levels, measured by ELISA, were reduced in psoriasis patients compared to controls, similar to our results; however, the difference was not significant, while significantly reduced levels of serum soluble PD-1 were detected in psoriasis patients compared to controls, though PD-L1 was not assessed in the study.

Moreover, a study done by Bartosińska et al. [32] showed significantly reduced absolute numbers and percentages of CD4 + PD-1 + and CD8 + PD-1 + T-cells in psoriasis patients compared to the controls like our study.

To the contrary of LP and psoriasis, and because of different comparable pathology, vitiligo patients have higher PD-1 levels on peripheral regulatory CD4 + and cytotoxic CD8 + T-lymphocytes. Also, PD-1 + mononuclear cells have been identified in peri-lesional vitiligo skin suggesting the role of PD-1 in disease immunopathogenesis. Based on these findings, the agonistic PD-1/PD-L1 drugs might inhibit the autoreactive T-lymphocytes [33].

Another study was done by Awad et al. [17]. who reported that PD-1 expression was detected in all marginal biopsies, being higher in lesional skin compared to nonlesional skin. The authors claimed that the higher levels of PD-1 in the marginal biopsies could be related not only to the increased infiltrates there but also to a lower CD4 + /CD8 + T-cells ratio, which is accompanied by more T-lymphocytes exhaustion and PD-1 expression.

In the current study, there was no significant correlation between LPSI with PD-1, PD-L1 (lesional and nonlesional) and duration of the disease, and there was no significant correlation between PD-1 and PD-L1 and the gender of the patients.

However, there was a statistically significant negative correlation between PD-1 and PD-L1 in the lesional and nonlesional skin and age of the patients, which is explained by the presence of cutaneous LP more commonly in older age groups (between the ages of 30 and 60 years) [34].

On the contrary, Shimada et al. [35] and Lee et al. [36] studied the T-lymphocytes senescence and intrinsic changes of T-cell signalling and reported that high levels of PD-1 expression in aged T-lymphocytes. These cells showed defective proliferation and cytokine production after T-cell receptor stimulation, and PD-1 expression is higher in old mouse T-cells.

Moreover, significantly higher PD-1 and PD-L1 levels were found in nonlesional skin in both short duration (≤ 2 months) and chronic (˃2 months) disease compared to lesional PD-1 and PD-L1 levels, however, no statistically significant differences were found upon comparing the short duration and chronic disease as regard the levels of both PD-1 and PD-L1 in either lesional or nonlesional skin of LP patients. These findings are in line with the forementioned detected study results, suggesting that lower levels of PD-1 and PD-L1 in lesional skin of LP patients could play a role in the pathogenesis of the disease, rather than affecting the chronicity of LP. However, to the best of our knowledge this is the first study to compare the levels of PD-1 and PD-L1 with cutaneous LP disease duration.

The small sample size, besides the inability to determine the type of cells expressing PD-1 and PD-L1, represent the main limitations of the current study. Therefore, further larger scale studies with immunohistochemistry are certainly needed.

## Conclusion

Significantly low levels of PD-1/PD-L1 in lesional and nonlesional skin of LP patients compared to controls explains that compromised PD-1/PD-L1 pathway may have a role in LP pathogenesis. Further larger-scale studies are needed to verify or negate this study’s findings.

Studies to evaluate the effect of various treatment options of LP on the tissue level of PD-1/PD-L1 are needed, using the flowcytometry or double staining method is suggested as methods to determine the cells expressing PD-1 and PD-L1.

Genetic polymorphism studies to search for polymorphisms in PD-1 and PD-L1 genes in LP patients are also suggested. Finally, further studies are needed to assess the therapeutic efficacy of PD-1/PD-L1 agonists in the treatment of LP.

### Supplementary Information

Below is the link to the electronic supplementary material.

Supplementary file1 (DOCX 256 kb)

## Data Availability

The data supporting findings of this study are available within the article. Raw data from which the findings of this study were obtained, are available from the corresponding author, upon request.
