# Supplementary material for: Assessment of PD-1 and PD-L1 tissue expression levels in lichen planus patients: a case–control study
Source: Arch Dermatol Res. 2024 Mar 2;316(3):97. doi: 10.1007/s00403-024-02838-z (PMC10908618; doi:10.1007/s00403-024-02838-z)
Supplement: Supplementary file 1 — Supplementary file1 (DOCX 256 kb) [file 403_2024_2838_MOESM1_ESM.docx]

**Calculation of LPSI:**

**Step 1: Assigning a body surface area factor**

Total involved body surface area is calculated using the Wallace rule of nines, the skip areas are excluded.

**Step 2: Lesion count and percentage**

Total number of lesions are counted followed by counting the total number of erythematous papules, violaceous papules, violaceous plaques, hyperpigmented hypertrophic papules and plaques and postinflammatory hyperpigmentation. Individual percentage of each of these lesions is determined.

**Step 3: Assigning area involving factor**

Area involvement factor is measured as per the above percentage for each morphological type of lesion. So, if percentage of a lesion is 0%–25% of total body surface area involved, its area involvement factor will be 1. Likewise for 26%–50%, 51%–75% and 76%–100%, area involvement factor will be 2, 3 and 4, respectively.

**Step 4: Multiplication factor**

Severity of the disease is assessed by natural history and morphology of lesions. Hypertrophic lesions are regarded as the most severe and are assigned the maximum value while postinflammatory hyperpigmentation is the treated inactive form, so, has the minimum value. Multiplication factor used for each morphological type of lesion is as follows:

• Hyperpigmented hypertrophic papules and plaques (Hp) – 4

• Violaceous flat plaques (Vpl) – 3

• Violaceous flat papules (Vp) – 2

• Erythematous papules (Ep) – 1

• Postinflammatory hyperpigmentation (PIH) – 0

**Statistical methods:**

Data were coded and entered using the statistical package for the Social Sciences (SPSS) version 28 (IBM Corp., Armonk, NY, USA). Data was summarized using mean, standard deviation, median, minimum and maximum in quantitative data and using frequency (count) and relative frequency (percentage) for categorical data. Comparisons between quantitative variables were done using the non-parametric Mann-Whitney test. For comparison of paired measurements within each patient the non-parametric Wilcoxon signed rank test was used. For comparing categorical data, Chi square (χ2) test was performed. Exact test was used instead when the expected frequency is less than 5. Correlations between quantitative variables were done using Spearman correlation coefficient. ROC curve was constructed with area under curve analysis performed to detect best cutoff value of markers for detection of cases. P-values less than 0.05 were considered as statistically significant.

**Laboratory work:**

**Human programmed cell death protein 1 (PD-1) and human programmed cell death ligand 1 (PD-L1) ELISA Kits**

**Catalog Number:** PD-1 (ELK4353), PD-L1 (ELK3055) 96 tests.

**PRINCIPLE OF THE ASSAY**

Test principle applied in this kit is Sandwich enzyme immunoassay. The microtiter plate provided in this kit has been pre-coated with an antibody specific to PD-1/PD-L1 Standards or samples are added to the appropriate microtiter plate wells then with a biotin-conjugated antibody specific to PD-1/PD-L1. Next, Avidin conjugated to Horseradish Peroxidase (HRP) is added to each microplate well and incubated. After TMB substrate solution is added, only those wells that contain PD-1/PD-L1, biotin-conjugated antibody and enzyme-conjugated Avidin will exhibit a change in color. The enzyme-substrate reaction is terminated by the addition of sulphuric acid solution and the color change is measured spectrophotometrically at a wavelength of 450nm + 10nm. The concentration of PD-1/PD-L1 in the samples is then determined by comparing the OD of the samples to the standard curve.

**DETECTION RANGE**

0.16 -10 ng/ml

**SENSITIVITY**

PD-1 (0.063 ng/ml), PD-L1 (0.056 ng/ml).

**SPECIFICITY**

This assay has high sensitivity and excellent specificity for detection of PD-1/PD-L1. No significant cross-reactivity or interference between human PD-1/PD-L1 and analogues was observed.

**PRECISION**

Intra-assay Precision (Precision within an assay): CV %< 8%

Three samples of known concentration were tested twenty times on one plate to assess.

Inter-assay Precision (Precision between assays): CV %< 10%

Three samples of known concentration were tested in forty separate assays to assess inter-assay precision.

**KIT components and storage:**

**Table:** KIT components and storage.

| Reagents | Quantity | | Storage condition | |  |
| --- | --- | --- | --- | --- | --- |
| Pre-coated microplate | 12 strips x 8 wells | | 4°C/ -20°C ( 6 months) | |  |
| Standard (lyophilized) | 2 | | 4°C/ -20°C ( 6 months) | |  |
| Standard Diluent Buffer | 20 Ml | | 4°C | |  |
| Biotinylated Antibody (100x) | 120 μL | | 4°C/ -20°C ( 6 months) | |  |
| Biotinylated Antibody Diluent | 12 ml | | 4°C | |  |
| Streptavidin-HRP(100x) | 120 μL | | 4°C/ -20°C ( 6 months) | |  |
| HRP Diluent | 12 ml | | 4°C | |  |
| Wash Buffer (25 x) | | 20 ml | | 4°C | |
| TMB Substrate Solution | | 9 ml | | 4°C (lucifuge) | |
| Stop reagent | | 6 ml | | 4°C | |
| Plate covers | | 2 | | 4°C | |
| Instruction manual | | 1 | | 4°C | |

**Special Explanation**

1. Please store the kit at 4°C if used up in one week.
2. If used for more than 1 week, store the Pre-coated Microplate, Standard, Biotinylated Antibody and Streptavidin-HRP at -20°C and all other reagents at 4°C according to the temperature indicated on the label.
3. Avoid repeated freezing and thawing.
4. Do not use the kit after the expiration date.
5. Please check whether all components are complete after opening the package.

All kit components have been formulated and quality control tested to function successfully as a kit. Do not mix or substitute reagents or materials from other kit, performance cannot be guaranteed if utilized separately or substituted.

**Materials required and not supplied**

1. Micro plate reader capable of measuring absorbance at 450 nm± 10 nm.
2. High speed centrifuge.
3. Electro-heating standing-temperature cultivator.
4. Absorbent paper.
5. Distilled or deionized water.
6. Single or multi-channel pipettes with high precision and disposable tips.
7. Precision pipettes to deliver 2μl to 1 ml volumes.

**Safety notes**

1. This kit is sold for lab research and development use only and not for use in humans or animals.
2. Reagents should be treated as hazardous substances and should be handled with care and disposed of properly.
3. Gloves, lab coat, and protective eyewear should always be worn, Avoid any skin and eye contact with Stop Solution and TMB. In case of contact, wash thoroughly with water.

**SAMPLE COLLECTION AND STORAGE**

. **Serum** Use a serum separator tube (SST) and allow samples to clot for two hours at room temperature or overnight at 4°C before centrifugation for 15 minutes at 1000 ×g (PD-1), 20 minutes at 1000 ×g (PD-L1) . Remove serum and assay immediately or aliquot and store samples at -20°C or -80°C. Avoid repeated freeze-thaw cycles.

. **Plasma** Collect plasma using EDTA, or heparin as an anticoagulant. Centrifuge for 15 minutes at 1000 x g, 2 - 8°C within 30 minutes of collection. Assay immediately or aliquot and store samples at -20°C or -80°C. Avoid repeated freeze-thaw cycles.

. **Tissue Homogenates** -The preparation of tissue homogenates will vary depending upon tissue type.

1. Tissues were rinsed in ice-cold PBS to remove excess blood thoroughly and weighed before homogenization.

2. Minced the tissues to small pieces and homogenized them in fresh lysis buffer (different lysis buffer needs to be chosen based on subcellular location of the target protein) (PBS can be used as the lysis buffer of most tissues) (w:v = 1:9, e.g. 900 uL lysis buffer is added in 100 mg tissue sample) with a glass homogenizer on ice (Micro Tissue Grinders woks, too).

3. The resulting suspension was sonicated with an ultrasonic cell disrupter till the solution is clarified.

4. Then, the homogenates were centrifuged for 5 minutes at 10000xg, Collection the supernatant and assay immediately or aliquot and store at <-20°C.

**CELL LYSATES** - Cells need to be lysed before assaying according to the following directions:

1. Adherent cells should be washed by cold PBS gently, and then detached with trypsin, and collected by centrifugation at 1000xg for 5 minutes (suspension cells can be collected by centrifugation directly).

2. Wash cells three times in cold PBS.

3. Cells were then resuspended in fresh lysis buffer with concentration of 10^7^ cells/mL. If it is necessary, the cells could be subjected to ultrasonication till the solution is clarified.

4. Centrifuge at 1500xg for 10 minutes at 2-8°C to remove cellular debris. Assay immediately or aliquot and store at <-20°C.

**Urine (for PD-L1) -**Aseptically collect the first urine of the day (mid-stream), voided directly into a sterile container. Centrifuge to remove particulate matter, assay immediately or aliquot and store at ≤-20°C.Avoid repeated freeze-thaw cycles.

**Saliva (for PD-L1) -** Collect saliva using a collection device or equivalent. Centrifuge samples for 15 minutes at 1,000×g at 2-8°C. Remove particulates and assay immediately or store samples in aliquot at ≤-20°C. Avoid repeated freeze/thaw cycles.

**Cell culture supernatants and other biological fluids (for PD-L1)** - Centrifuge samples for 20 minutes at 1000×g. Collect the supernatant and assay immediately or store samples in aliquot at -20°C or -80°C for later use. Avoid repeated freeze/thaw cycles.

**NOTES**

1.Samples to be used within 5 days may be stored at 4°C, otherwise samples must be stored at -20°C (≤1 month) or -80°C (≤2 months) to avoid loss of bioactivity and contamination. Avoid repeated freeze/thaw cycles.

2. Sample hemolysis will influence the result, so hemolytic specimen should not be used.

3. When performing the assay, bring samples to room temperature.

4. If the concentration of the test material in your sample is higher than that of the standard product, please make the appropriate multiple dilution according to the actual situation (it is recommended to do preliminary experiment to determine the dilution ratio

**Summary**

- - 1. After the kit is equilibrated at room temperature, add 100μL of standard working Buffer (gradually diluted according to the instructions) or 100μL of sample to each well, incubate at 37°C for 80 minutes.


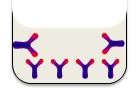

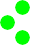

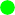

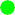

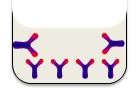

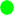

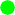

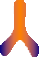

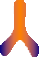

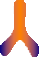

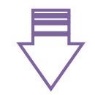

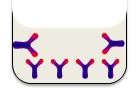

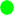

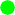

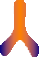

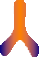

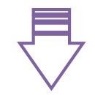

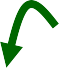

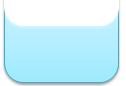

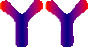

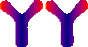

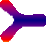

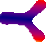

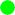

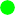

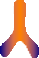

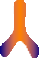

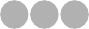

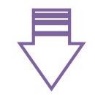


- - 1. Discard the liquid in the plate, add 200μL of Wash Buffer to each well, and wash the plate 3 times. After spin-drying, add 100μL Biotinylated Antibody working solution to each well,incubate at 37°C for 50 minutes.
    2. Discard the liquid in the plate, add 200μL Wash Buffer to each well, and wash the plate 3 times. After drying, add 100μL Streptavdin-HRP working solution to each well, incubate at 37°C for 50 minutes.


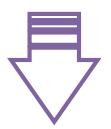


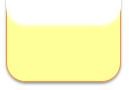

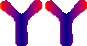

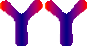

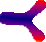

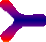

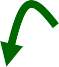

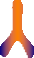

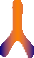

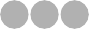


- - 1. Discard the liquid in the plate, add 200μL Wash Buffer to each well, and wash the plate 5 times. After spin-drying, add 90μL TMB to each well,incubate at 37°C for 20min.
    2. Add 50μL stop solution to each well, read plate at 450nm immediately, calculation of the results.

**REAGENT PREPARATION**

1. Bring all kit components and samples to room temperature (18-25°C) before use.

2. If the kit will not be used up in one time, please only take out strips and reagents for present experiment, and save the remaining strips and reagents as specified.

3. Dilute the 25x wash buffer into 1x working concentration with double steaming water.

4. **Standard working solution**-Reconstitute the Standard with 1.0mL of Standard Diluent, kept for 10 minutes at room temperature, shake gently(not to foam). The concentration of the standard in the stock solution is 10 ng/mL. Please prepare 7 tubes containing 0.5mL Standard Diluent and use the diluted standard to produce a double dilution series according to the picture shown below. Mix each tube thoroughly before the next transfer. Set up 7 points of diluted standard such as 10 g/mL, 5 ng/mL, 2.5 ng/mL, 1.25 ng/mL, 0.63 ng/mL, 0.32 ng/mL, 0.16 ng/mL, and the last EP tubes with Standard Diluent is the blank as 0 ng/mL. In order to guarantee the experimental results validity, please use the new standard solution for each experiment

5. **Biotinylated Antibody and Streptavidin-HRP:** Briefly spin or centrifuge the stock Biotinylated Antibody and Streptavidin-HRP before use. Dilute them to the working concentration 100-fold with Biotinylated Antibody Diluent and HR Diluent, respectively.

6. **TMB substrate** - Aspirate the needed dosage of the solution with sterilized tips and do not dump the residual solution into the vial again.

**NOTES**

1. Disposable pipette tips, flasks or glassware are preferred, reusable glassware must be washed and thoroughly rinsed of all detergents before use.

2. Bacterial or fungal contamination of either samples or reagents or cross-contamination between reagents may cause erroneous results.

3. All residual washing liquid must be drained from the wells by efficient aspiration or by decantation followed by tapping the plate forcefully on absorbent paper. Never insert absorbent paper directly into the wells.

4. If crystals have formed in the Wash Solution concentrate (25x), warm to room temperature and mix gently until the crystals are completely dissolved.

5. Prepare standards within 15 minutes before assay. This standard can only be used once.

6. The TMB solution is light sensitive. Avoid prolonged exposure to light. Also, avoid contact of the TMB solution with metal to prevent color development. Warning MB is toxic avoid direct contact with hands. Dispose of properly. If a dark blue color develops within a few minutes after preparation, this indicates that the TMB solution has been contaminated and must be discarded.

7. When pipetting reagents, maintain a consistent order of addition from well-to-well. This will ensure equal incubation times for all wells. Dispense the TMB solution within 15 minutes following the washing of the microtiter plate.

8. It is highly recommended to use the remaining reagents within 1 month provided this is prior to the expiration date of the kit. For the expiration date of the kit, please refer to the label on the kit box.

**SAMPLES PREPARATION**

1. Equilibrate all materials and prepared reagents to room temperature prior to use. Prior to use, mix all reagents thoroughly taking care not to create any foam within the vials.

2. The user should calculate the possible amount of the samples used in the whole test. Please reserve sufficient samples in advance.

3. Please predict the concentration before assaying. If values for these are not within the range of the standard curve, users must determine the optimal sample dilutions for their particular experiments.

**ASSAY PROCEDURE**

1. Determine wells for diluted standard, blank and sample. Prepare 7 wells for standard, 1 well for blank. Add 100 μL each of standard working solution (read Reagent Preparation), or 100 μL of samples into the appropriate wells. Cover with the Plate sealer. Incubate for 80 minutes at 37°C.

2. Remove the liquid of each well. Aspirate the solution and wash with 200 μL of 1× Wash Solution to each well and let it sit for 1-2 minutes. Remove the remaining liquid from all wells completely by snapping the plate onto absorbent paper. Totally wash 3 times. After the last wash, remove any remaining Wash Buffer by aspirating or decanting. Invert the plate and blot it against absorbent paper.

3. Add 100 μL of Biotinylated Antibody working solution to each well, cover the wells with the plate sealer and incubate for 50 minutes at 37°C.

4. Repeat the aspiration,wash process for total 3 times as conducted in step 2.

5. Add 100 μL of Streptavidin-HRP working solution to each well, cover the wells with the plate sealer and incubate for 50 minutes at 37°C.

6. Repeat the aspiration,wash process for total 5 times as conducted in step 2.

7. Add 90 μL of TMB Substrate Solution to each well. Cover with a new Plate sealer. Incubate for 20 minutes at 37°C (Don't exceed 30 minutes). Protect from light. The liquid will turn blue by the addition of TMB Substrate Solution.

8. Add 50 μL of Stop reagent to each well. The liquid will turn yellow by the addition of Stop reagent. Mix the liquid by tapping the side of the plate. If color change does not appear uniform, gently tap the plate to ensure thorough mixing. The insertion order of the Stop reagent should be the same as that of the TMB Substrate Solution.

9. Remove any drop of water and fingerprint on the bottom of the plate and confirm there is no bubble on the surface of the liquid. Then, run the microplate reader and conduct measurement at 450 nm immediately.

**CALCULATION OF RESULTS**

Average the duplicate readings for each standard, control, and samples and subtract the average zero standard optical density. Construct a standard curve with the Human PD-1/PD-L1 concentration on the y-axis and absorbance on the x-axis, and draw a best fit curve through the points on the graph. If samples have been diluted, the concentration read from the standard curve must be multiplied by the dilution factor. Using some plot software, for instance, curve expert.
